# Thermodynamic characteristics of the aliphatic polyamide crystal structures: Enhancement of nylon 66α, 610α and 77γ polymers

**DOI:** 10.1016/j.heliyon.2023.e21042

**Published:** 2023-10-16

**Authors:** Ali F. Al-Shawabkeh

**Affiliations:** Department of Scientific Basic Sciences, Faculty of Engineering Technology, Al-Balqa Applied University, Amman 11134 Jordan

**Keywords:** Polyamides, Nylons, Thermodynamics, Materials studio, Molecular dynamics

## Abstract

Despite the polymer industry's reliance on nylon polymers, numerous questions remain about their crystal structures, modeling, and other features. This work discusses the thermodynamic properties and molecular modeling of a polyamides nylon 66α, 610α, and 77γ crystal structure systems for use in various electronics and Nano-devices that feature distinct properties such as exceptional optoelectronic properties at a low cost compared to other structures. This study looked at the crystal structure of a linear polyamide chain made up of repeating units. The influence of the thermal expansion coefficient and thermodynamic parameters on crystal structures' characteristics at different temperatures has previously been explored. The findings of this study demonstrate, on the one hand, the influence of the amorphous phase on the final thermodynamic characteristics of semi-crystalline polymers and, on the other hand, pave the way for greater improvement in the durability of these polymers by increasing their crystalline features. The values of the thermodynamic parameters for nylon 66α, 610α and 77γ such as enthalpy (ΔH_Exp_.) were 35.08, 40.25, and 1.44 kJ/mol, entropy (ΔS_Exp._) 113.75, 128.84, and 15.10 J/mol-K, free energy (ΔG_Exp._) was −44.57, −46.62, and −6.86 kJ/mol, respectively. When the nylon data is compared, the nylon 610α exhibits a significantly higher free energy, at high temperatures, the process is spontaneous and exergonic, making it a potentially viable material for use as fibers and engineering thermoplastics.

## 1 Introduction

In polymer research, polymer product processing is a hotly disputed topic. The solidification method is an important component in processing because of its impact on structures, which typically affect product performance. Semicrystalline polymers [1] are the most commonly made, with a microstructure consisting of crystalline and amorphous phases. The complex combination of various phases in a semicrystalline polymer complicates experimental investigation of individual phases [2]. Because of its good mechanical, thermal, and chemical properties [3]. Polyamide (PA), a significant semicrystalline polymer, is employed in various industries, including military equipment [4], aerospace materials [5], insulation, and textile materials [6,7]. Thermoplastic polyamides offer excellent mechanical characteristics, thermal properties, corrosion resistance, chemical inertness, and abrasion resistance, making them highly demanded polymers in various industrial sectors [8,9]. Because of their exceptional mechanical and thermal qualities, aliphatic polyamides were the first synthetic and semicrystalline polymers to be used as fibers and engineering thermoplastics [10]. Nylons are being used in a variety of applications, most notably in the textile and automotive industries. Thermoplastic polyamides have recently been investigated in the literature to improve the manufacturing process [[11], [12], [13], [14]].

The drying stage before processing, however, is a crucial step in PA processing because components may develop with different defects induced by the presence of water in the material [15] since PA is hygroscopic.

Crystal structure and structural modification play critical roles in material characteristics during processing [16,17]. Some researchers have thoroughly investigated crystalline phase transitions during heating or cooling [[18], [19], [20]]. Mechanical properties are commonly linked to their deforms, which are mostly plastically yield stress. The solidification technique primarily influences the thermodynamic state, i.e., aging, in the case of glassy polymers; it was shown that aging increases as the cooling rate during solidification lowers, and therefore, the yield stress increases [21]. Semicrystalline polymers are more challenging; pressure and cooling rate can have a significant impact on material morphology, such as crystallinity and lamellar thickness [[22], [23], [24]]. Individual phase properties, on the other hand, can be calculated. Polyamide (PA), a common raw material fiber used in textiles and plastics, is made from dicarboxylic acids and diamines (AABB type) or ω-aminocarbonxylic acids (AB type) [25,26]. PAs are composed of sheets of fully extended planar chains distinguished by a group of “CONH”. The inter-strand and intra-strand hydrogen bonding of PAs dominates the regular arrangement of macromolecules within crystals [27].

The triclinic α-crystal structure of PA 66 may convert into the pseudo γ-hexagonal-crystal structure during heating, in a process named the Brill transition [[28], [29], [30]]; the corresponding transition temperature is named the Brill transition temperature (T_B_) [[31], [32], [33], [34], [35]]. Generally speaking, the T_B_ will change with the grain size and state, and sometimes even exceeds the melting point (T_m_) [36,37]. Materials having functional groups like C=O, OH, COOH, F, NH_3_, etc. depend on hydrogen bonding, a mainstream, non-covalent interaction [38]. Hydrogen bond bridges are critical in the crystallization of polyamide structures. Because of thermodynamic driving factors, Li et al. (2020) [39] observed that high hydrogen bond density favors fast crystallization at higher temperatures. Until recently, high-performance polymers such as polyamides (PAs) were solely generated from oil; however, new methods and research have succeeded in generating them from monomers that are both totally and partially renewable [40,41].

Polyamides often produce immiscible blends because the high molar mass of polymers reduces the beneficial entropic contribution to the free energy of mixing. Therefore, insignificant adverse enthalpy of mixing values causes a phase separation. Nylon 66, nylon 610, and nylon 77 are the aliphatic polyamides used in fabrics, carpet, hydraulic and pneumatic hoses, optical fiber sheathing, powder coating, and other durable goods applications [9]; as a result, the development of polyamides from renewable resources - one of the largest industrial scale engineering plastics of great significance - is crucial. Despite the importance of nylon polymers in the industry, there are numerous uncertainties concerning their crystal structures, and other properties. Nylon polymers are partially crystalline, but reliable experimental data on their ordered regions is difficult to obtain. Understanding the kinetics of crystallization, degree of crystallinity (X_c_), and thermal characteristics such as melting temperature (T_m_) and glass transition temperature (T_g_) are critical in selecting polymer and composite processing parameters [42]. The crystallization rate of semicrystalline polymers like PA is strongly affected by temperature and occurs between the T_g_ and T_m_ of the material, therefore knowing these temperatures is critical. High-performance thermoplastics' (X_c_) degree of crystallinity directly affects the chemical and mechanical properties of the polymer. While the amorphous phase impacts energy absorption, the crystalline phase improves stiffness and tensile strength [43].

The structure of these commercial nylons typically consists of a stack of structures made up of hydrogen-bonded molecular chains that can be parallel, anti-parallel (such as even nylons), or both (such as odd nylons), and have a molecular conformation that is almost entirely trans. The anti-parallel configuration was thought to be advantageous for odd nylons [44]. In contrast to the copolymerization of co-monomers, which alters the density of H-bonds between macromolecular chains having a functional group that can form hydrogen bonds, the efficient hydrogen bond interaction is the weak connection between the polyamide chain [45]. Variations in H-bond length in the sample are also related to structural changes. Lack of H-bond length and density in PA66 in its amorphous state. The OH–O bond separation distance and density are only studied at different temperatures in low molecular weight systems [46,47].

Both PA66 and PA610 have melting points (T_m_) less than 280 °C, short-term service temperatures less than 250 °C, and long-term service temperatures less than 220 °C, making them unsuitable for use in sectors requiring high-temperature resistance. That is the market for semicrystalline heat-resistant polyamides (HPAs) with T_m_ greater than 280 °C, or at least 270 °C as defined by industry. The market demand for HPAs has rapidly increased over the past ten years in tandem with rising plastic requirements in the electronic and electrical, automotive, aerospace, military, chemical, and other industries [48]. These HPAs must also have good performance and significantly improved heat-resistant temperature. The automotive sector is responsible for 35 % of PA consumption, the largest PA consumer [49]. Amorphous nylon 66 can be described from about 50 K to the glass transition temperature, T_g_ (=323 K), by the same vibrational C_p_ [50,51]. The equilibrium melting temperature, T°_m_, for nylon 6.6 is taken to be 574 K and the heat of fusion of a fully crystalline sample to be 57.8 kJmol^-1^ based on extensive discussions of data from a wide range of literature [52,53]. Typically, reverse engineering and numerical simulation techniques are used to accurately forecast the mechanical behavior of the processed elements in the application. Reliable lightweight structures are crucial for the application of this strategy, albeit [54,55].

The goal of the ongoing research is to address the problem using traditional thermodynamic techniques, which is made possible by the ability to calculate the thermodynamic quantities ΔH, ΔS, and ΔG. Unlike most organic processes, which involve the interaction of neutral molecules, the synthesis of an amide involves the interaction of electrically charged groups. The behavior of the crystal transition and its relationship with thermodynamic parameters of polyamides have recently been the subject of extensive study; however, the correlational research on semi-crystalline nylons is still lacking. This characteristic is crucial for the energy change in nylon structures. The effects of temperature on nylon 66 α, 610 α, and 77 γ crystal structure and crystallinity transition behavior are extensively explored in this work, and the findings will be crucial in helping to create high-performance aliphatic polyamides and broaden their applications.

## 2 Materials and methods

### 2.1 Linear PA chain model

**Fig. 1:**
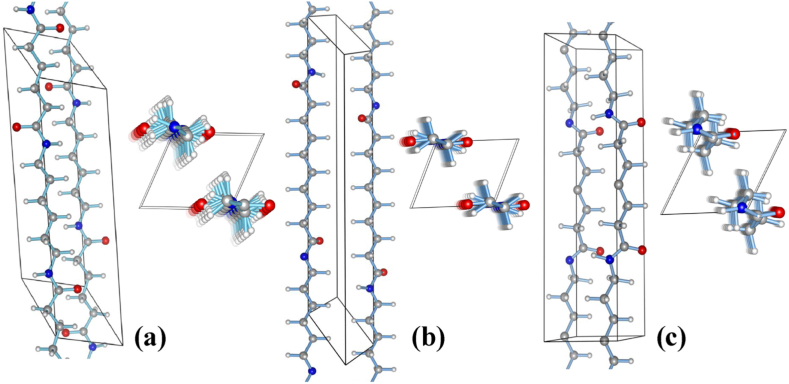
Schematic representation crystal structures: (a) nylon 66α (b) nylon 610α (c) nylon 77γ. *Color code*: *nitrogen*, *blue*; *oxygen*, *red*; *carbon*, *gray*; *hydrogen*, *white*.

Thermal methodology includes a lot of relevant and significant information about the states, critical points, and other thermodynamic characteristics of materials and molecules. For our investigation, we looked into the volume, temperature, structural makeup, and overall energy of nylon polymers. For the purpose of figuring out the melting point and glass transition temperature, particle atoms with different chain lengths were investigated. This article investigates the crystal structure of a repeating linear polyamide chain (Fig. 1). A repeat unit of the initial monomers was initially modeled using the Materials Studio program and the Reactive Force-Field (ReaxFF) analysis tool in order to create a linear polyamide chain.

It is difficult to build a model that adequately depicts the repeat unit since the atomic arrangement shows that two groups of monomers were covalently bound to the amino group of *m*-phenylenediamine (MPD) monomers. Consequently, MPD monomers' amino group is bonded to amide bonds monomers with the corresponding chemical formula. Table 2 displays the nylon polymers' material characteristics. Thus, the monomers groups of nylon 66 poly (hexamethylenediamine-*co*-adipic acid) (C_12_H_22_N_2_O_2_)_n_, nylon 610 poly (hexamethylene sebacamide) (C_16_H_30_N_2_O_2_)_n_, and nylon 77 poly (heptamethylene pimelamide) (C_14_H_26_N_2_O_2_)_n_ compose the generated single linear PA chain [56].

### 2.2 Types of Nylon

The following is the naming convention for unsubstituted nylon: Nylon-n is the polymer generated when the monoacid [NH_2_-(CH_2_)n-1-C(O)(OH)] is polymerized to form (1). It is labeled as nylon m,n when formed via the condensation of the diamine [H_2_N-(CH_2_)m-NH_2_] and the diacid [C(O)(OH)-(CH_2_)n-2-C(O)(OH)]. The most important nylons in terms of the market are nylon 66 and nylon 6, which are used in textiles and carpet fibers [57]. The crystal structures of nylon 66α, 610α, and 77γ are all identical. Fibers of these two polyamides often comprise two distinct crystalline forms, and, which are different packing's of geometrically similar molecules; the form makes up the majority of most fibers. The dimensions of simulated nylon polymer unit cells are displayed in Table 1.

**Table 1:** Unit cell dimensions of simulated polyamide nylons.

Polyamide	a [Å]	b [Å]	c [Å]	α [deg]	β [deg]	γ [deg]
**Nylon 66α**	4.90	5.40	17.20	48.50°	77.00°	63.50°
**Nylon 610α**	4.95	5.40	22.40	49.00°	76.50°	63.50°
**Nylon 77γ**	4.82	18.95	4.82	90.00°	60.00°	90.00°

In the case of the 66 polymers, fibers with no detectable amount of the β form were obtained. Unit cell dimensions and the indices of reflexions for the α form were determined by trial. The unit cell of a form is triclinic, for the 66 polymer: a = 4.90 Å, b = 5.40 Å, c (fibre axis) = 17.20 Å, α = 48.50°, β = 77.00°, γ = 63.50°; for the 610 polymer: a = 4.95 Å, b = 5.40 Å, c (fibre axes) = 22.40 Å, α = 49.00°, β = 76.50°, γ = 63.50°; and for the 77 polymer: a = 4.82 Å, b = 18.95 Å, c (fibre axes) = 4.82 Å, α = 90.00°, β = 60.00°, γ = 90.00°. One chain molecule passes through the cell in both structures 66α and 610α.

The relative strengths of the reflexions were interpreted to yield the atomic coordinates in crystals. The oxygen atoms appear to be somewhat out of the chain's plane; however, the chains are planar or very nearly so. Hydrogen connections between the C=O and NH groups connect the molecules to form sheets. The α arrangement is produced by merely packing these planar chains of molecules.

### 2.3 Crystal structures

The crystal structures discovered for nylons are classified into two types: (1) α and β phases (which include even-even nylons 66 and 610), and (2) γ phase (this includes even nylons from −8 up and the even-odd, odd-even and odd-odd nylons) [58]. Planar sheets of hydrogen-bonded chains are shown in Fig. 1-a, and 1-b, piled on top of one another and offset by a predetermined distance in the chain direction. The β phase lacks distinction, most likely as a result of a slight alteration to the α phase. This form has no distinct crystal structure and serves no useful purpose. The γ phase consists of pleated methylene unit sheets with hydrogen bonding between sheets rather than inside sheets. The sheets are straightforward to identify in the α phase because the methylene spacers are almost coplanar with the amides, which are H-bonded to amides from adjacent chains. As a result, when the methylene spacers are twisted with respect to the amide planes, we refer to the methylene spacers as the sheets and consider the amides to be H-bonded to chains in adjacent sheets rather than inside the same sheet in the γ phase (See Fig. 1-c). The amide-to-methylene dihedrals are near trans (164–168°) in α form vs almost perpendicular to the peptide plane (126°) in γ form, which is the primary structural difference between the α and γ forms. Typically, axial tension can change the γ form into the α form [59,60]**.**

The simulation box (xyz vectors) of the equilibrated system is then increased, and additional PA chains are inserted. In this structure of PA chains, energy minimization (EM) and equilibration were repeated. This process continued until the energy of the system was no longer possible to be minimized. This allowed for the successful insertion, energy minimization, and equilibration of a total of 128 linear PA chains. This strategy allowed careful control of the density of the system to reach a target dehydrated PA membrane density value of 1.06 gcm^−3^ (Mi et al., 2007) [61]. The average density for nylon polymers was found to be (1.06–1.23 gcm^−3^), near the experimental value of 1.06 gcm^−3^ for dehydrated PA membrane and in fair agreement with previous simulation works (Gao et al., 2015; Xiang et al., 2013) on fully linear PA membrane [62,63].

The range of values discovered throughout this review are shown in Table 2. Nylons have a melting point that ranges from approximately 196 to 280 °C. Nylon 77γ had the lowest melting point temperature, while nylon 66α had the highest reported value. Nylon 610α was found to be in the 226–232 °C range. In polyamides from diamines and di-basic acids, the even numbers of CH_2_ series 66α, 610α have the highest melting points, while the odd numbers of di-basic acids series 77γ have the lowest. Hydrogen bonds, which significantly boost the cohesive forces between the molecules, are to blame for this. It should be noted that the relationship between polyamide series' density and CH_2_ number bears striking similarities to that between melting point and CH_2_. A molecule with even number of CH_2_ groups are more likely to pack together in a particular way than a molecule with an odd number. This is due to the fact that the relationship between density and CH_2_ or CH_2_ reveals the molecular packing properties.

**Table 2:** Material properties of the studied polyamide nylons 66α, 610α, and 77γ.

Property	Nylon 66α	Nylon 610α	Nylon 77γ	Test method
**Molecular Weight***[g.mol*^*−*^*^1^]*	226.32	282.40	254.21	ASTM D6474
**Density***[g.cm*^*−*^*^3^]*	1.23	1.18	1.06	ASTM D1505
**Specific Gravity***[g.cm*^*−*^*^3^]* **Melting Temperature***[°C]*	1.14 265–280	1.52 226–232	1.20 196–214	ASTM D792 ASTM D3418
**Glass Transition Temperature***[°C]*	50–60	45–55	40–55	ASTM E1356
**Thermal Expansion Coefficient***[K*^*−*^*^1^]*	7.0 × 10^−5^	1.6 × 10^−5^	3.5 × 10^−5^	ASTM D696
**Specific Heat Capacity***[J.g*^*−*^*^1^°C*^*−*^*^1^]*	1.47	1.59	1.09	ASTM E1269
**Thermal conductivity***[W.m*^*−*^*^1^K*^*−*^*^1^]*	0.23	0.25	0.24	ASTM C177

The quantity of polyamide CH_2_ groups that are contained in a particular molecule determines the structure of a nylon. The impact of strong intermolecular contacts on the rheology of polyamides will be investigated, with the introduction of various interacting groups (such as ionic and H-bonds) with the aim of enhancing the strength and density of the connection between polymer chains.

According to some researchers [64,65], this peak was caused by the melting of microcrystals that had developed in the amorphous region. Structure modifications during post-crystallization can take the form of chain rearrangements brought on by hydrogen bond breaking, tie-molecule relaxation, and accelerated chain motion [66]. According to Shu et al., as the chain motion increases, mobile chains in the amorphous zone might interact with the crystallites, increasing the crystallinity [67].

This study uses simulated molecular dynamics to measure the thermodynamic characteristics of polyamide directly. Molecular entanglements and physical crosslinks brought on by crystallinity could cause a MW to fracture a polymer. Data on polymers and temperature were collected simultaneously. The thermodynamic properties and behavior of polyamide polymers are described by a model that uses five factors, including the coefficient of thermal expansion.

## 3 Results and discussion

### 3.1 Molecular dynamics

MD is a computational method that is used to simulate how a system of particles' locations, velocities, forces, and trajectories change over time. It provides a physical foundation for comprehending the dynamics, interactions, processes, and structure of some materials and biomolecules (Karplus and McCammon, 2002) [68]. The estimated empirical interaction potential for the particles is necessary for MD (the force-field). A force-field is a functional expression of the total potential energy of the system's particles that includes intramolecular (bond stretching, angle bending, dihedral, and incorrect torsions) and intermolecular (van der Waals and Coulombic interactions) components. A set of parameters must also be included, which are often derived via experiments, by quantum mechanical calculations, or a combination of the two. Due to the covalent bonding of two monomer groups to the amino group of MPD monomers, amide bonds were formed, as evidenced by the atomic arrangement in the repeat unit.

#### 3.1.1 Potential energy

Polyamides are made up of the amide groups (linkage of amine with the carboxylic acid group) that engage alongside linear alkane chains. Hydrogen bonding between amide groups of neighboring chains performs a most important function in the surface properties of polyamides. A coarse-grained (CG) model is utilized to represent the component monomers and subsequent oligomers in order to minimize the number of force computations. The goal of this method is to reduce the number of internal degrees of freedom while producing a representation that polymerizes in a way that the bond lengths and molecule conformations match those of a realistic atomic structure.

**Fig. 2:**
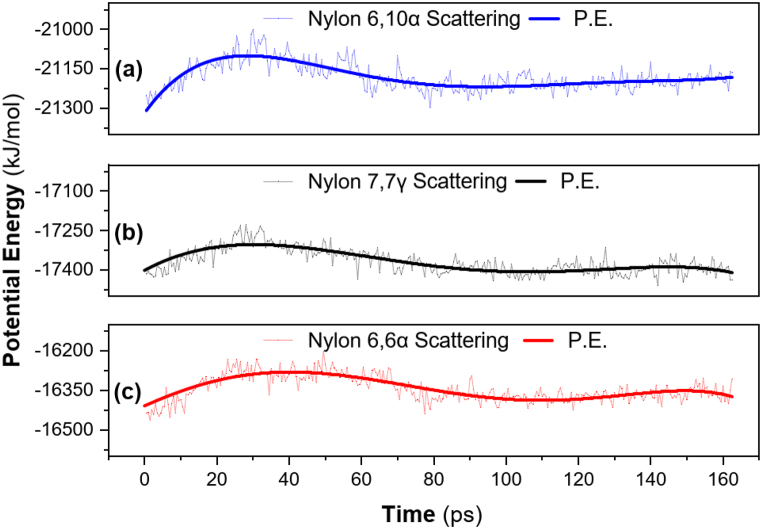
Potential energy versus time for nylons: (a) nylon 66α (b) nylon 610α (c) nylon 77γ.

Comparing the data for nylon 66α with that for nylon 610α and nylon 77γ (as shown in Fig. 2, and Table 3). It is evident that the nylon 66α exhibits substantially higher potential energy about −16.280 × 10^3^ kJmol^−1^, and the change in potential energy equilibrium state for all structural systems after 90 ps. For chain rotation, thermal energy crosses the threshold of potential energy. Viscoelastic characteristics change quickly with time and temperature changes close to the temperature of vitrification transition [69]. Since the potential energy is periodic, a translation of the crystal lattice has no impact on it. Therefore, its plane wave expansion will only take into account plane waves that have the periodicity of the lattice. The non-bonded potential should remain in its original shape for all atoms, according to Damewood et al. (1990), as this avoids the need to choose which atoms are unique H-bonded atoms. They offered a technique to parameterize H-bond parameters using experimental (and/or ab initio) data and the standard vdW potentials for the other atoms [70]. However, rather than focusing on the entire potential energy curve, all of these approaches concentrate on the binding energy and the equilibrium bond length of the H-bond.

**Table 3:** Potential energy values for the polyamide nylons.

Nylon	***Potential Energy (10^3^) [kJmol^−1^]***		
	Min.	Max.	Mean
66 α	−16.408	−16.280	−16.342
610 α	−21.308	−21.100	−21.181
77 γ	−17.409	−17.303	−17.367

**Fig. 3:**
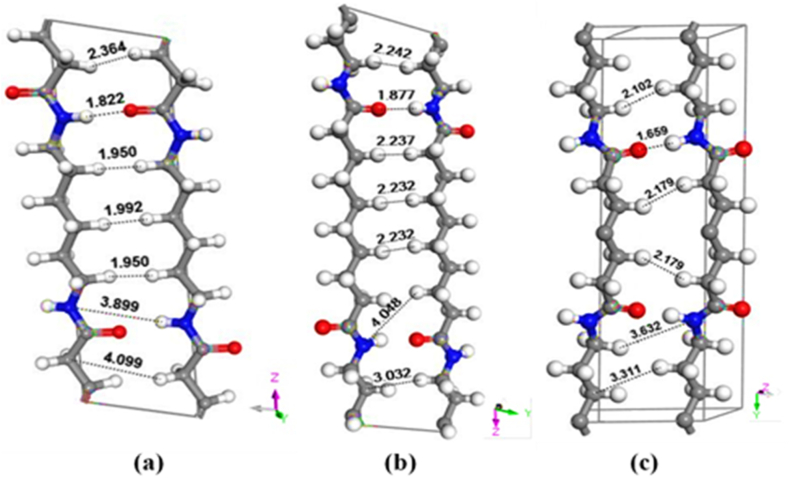
Bond distance for polyamides: (a) nylon 66α (b) nylon 610α (c) nylon 77γ.

As illustrated in Fig. 3(a–c) for the α and γ forms of the nylon 66 and nylon 77 structures, the left side shows the view of the hydrogen bonding locations and the right n side shows the view down the chain axis. In the R form of nylon 66, the neighboring chains are anti-parallel, and within the same sheet, the adjacent chains form hydrogen bonds (bisecting the CH_2_ angles). In neighboring sheets of nylon 66, when the chains are parallel, hydrogen bonding occurs between the chains. The average H-bonding distances for nylon 66 α, nylon 610 α, and nylon 77γ are 2.064 Å, 2.236 Å, and 2.153 Å, respectively, while the shortest H-bonding is 1.950 Å, 2.232 Å, and 2.102 Å. The shortest non-bonded distance between alkyl H atoms on distinct chains, according to Siddharth (1996), is 2.14 Å, which is significantly less than the 2.45 Å in polyethylene. In contrast, the smallest length in the structure is 2.47 Å. This demonstrates that hydrogen bonding causes undesirable CH_2_**⋯**CH_2_ interactions in the α form by squeezing the chains together [71]. The hydrogen bonding distance of N::O from model structures for nylon 66α, nylon 610α, and nylon 77γ, are 2.781 Å, 2.796 Å, and 2.768 Å, respectively.

**Fig. 4:**
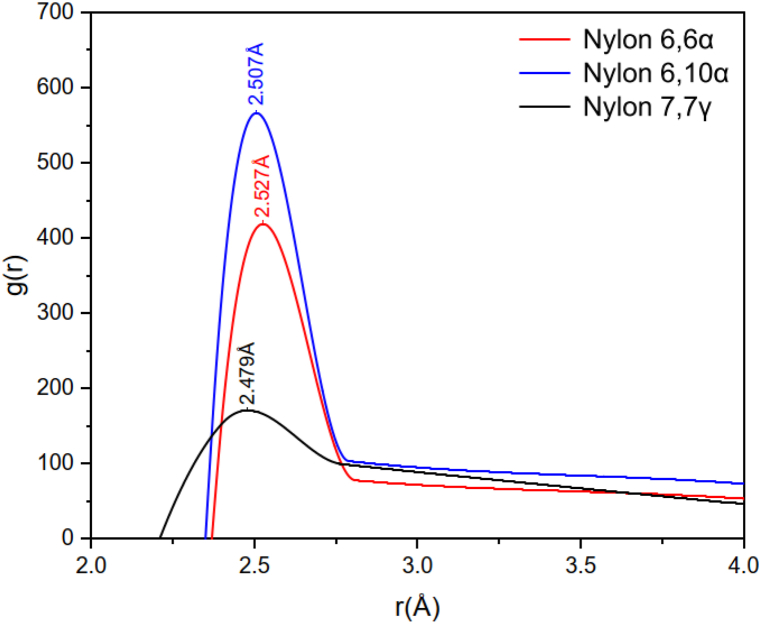
Radial distribution function for N::O bonding of Nylon 66 α, Nylon 610 α, and Nylon 77 γ structures.

As seen in Fig. 4 the demonstrated N::O bonding distances in nylon 66 alpha, nylon 61 alpha, and nylon 77 gamma verified by the radial distribution function in simulations are 2.507 Å, 2.527 Å, and 2.479 Å, respectively. This finding is consistent with the value 2.980 Å published by Malta et al. (1979) [72]. In each sheet, all of the amide groups connected by NH**⋯**O hydrogen bonds are coplanar. The directions of the NH**⋯**O hydrogen bonding are indicated by the broken lines in Fig. 3. The molecule is satisfactorily packed, with appropriate vdW gaps between succeeding sheets. It should be highlighted that the current examination shows the pleated sheet structure as mentioned above for polyamides with the typical amounts of CH groups.

#### 3.1.2 Kinetics energy

In order for entangled PA chains to crystallize, kinetic thermodynamics is essential. According to the homogeneous nucleation theory of crystal growth, liquid fluctuations at equilibrium allow for the creation of critical size nuclei that are stable for growth. While analogous shifts in entangled nylons at their equilibrium T_m_ are relatively unusual, they do happen for low-molar-mass molecules at their melting temperature. The free energy for polymer segment alignment into critical size nuclei decreases below T_m_. The opposite effect of viscosity increasing at lower temperatures is a reduction in the occurrence of the necessary variations. Optimal crystallization rates between T_g_ and T_m_ are produced by the interaction of these two factors [73].

**Fig. 5:**
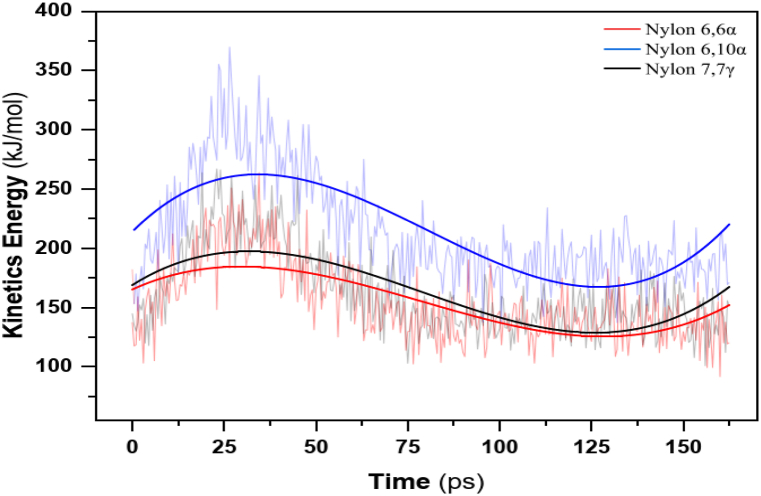
Kinetics energy with time for nylons: 66α, 610α, and 77γ.

Fig. 5 shows the harmonic oscillation behavior of kinetic energy with time for nylon polymers. By reducing particle mobility and condensing system volume, this harmonic cause pulls the particles closer together, raising the polyamide membrane density. The kinetic energy sinusoidal wave varies with time for nylon 66α, 610α, and 77γ from 125.643 to 184.576, 167.427 to 262.364 kJmol^-1^, and from 128.902 to 197.360 kJmol^-1^ after fitting high harmonics for the polymers. The relative mean values are 154.922, 214.925, and 163.109 kJmol^-1^, respectively. Based on these findings, nylon 610α has the maximum kinetic energy of 214.93 kJmol^-1^.

#### 3.1.3 Crystal structure intermolecular energy

The overall intermolecular energy of a molecular structure system can be represented as the sum of all adopted interactions. This is done by combining the energy components for all relevant atom pair interactions, which are encapsulated in Equation (1):

(1) $$E_{\text{system}}=E_{\text{bond}}+E_{\text{over}}+E_{\text{angle}}+E_{\text{tors}}+E_{vdW}+E_{\text{Coulomb}}+E_{\text{Specific}}$$

Where: E_bond_ is the energy involved with the formation of bonds between atoms. The energies E_angle_ and E_tors_ are linked with three-body valence angle strain and four-body torsional angle strain, respectively. E_over_ is an energy penalty that prevents atoms from over coordination. Regardless of connectivity or bond order, the contributions of electrostatic and dispersive forces are computed between all atoms. E_Specific_ refers to phrases that are system-specific and uncommon [74].

The structural energy related to the formation between nylon atoms are presented in Table 4, the total intermolecular energies of nylon 66α, 610α, and 77γ are −16306.06, −21164.82, and −17437.79 kJmol^-1^, respectively.

**Table 4:** Intermolecular structural energies associated with atoms of polyamide nylons.

Energy *[kJmol*^*−*^*^1^]*	Nylon 66α	Nylon 610α	Nylon 77γ
**Over-/Under coordination energy**	−166.970	−165.002	−262.261
**Bond energy**	−23405.600	−30509.400	−24542.400
**Conjugation energy**	−119.294	−164.504	−117.971
**Hydrogen bond energy**	−0.322	−2.269	−13.105
**Lone pair energy**	−1.8 × 10^−9^	−2.3 × 10^−9^	−2.2 × 10^−9^
**Coulomb energy**	−690.596	−724.053	−823.962
**Penalty dbl bond energy**	2.219	2.148	3.061
**Torsion angle energy**	314.713	335.442	271.970
**Valence angle energy**	877.645	1115.996	829.342
**VdWaals energy**	6561.746	8607.303	6786.820
**Charge energy**	320.395	339.520	430.717

The high surface energy of nylons, which is primarily brought about by H-bond interactions, restricts the formation of voids in these materials. Inherently self-healing processes take place in nylon materials because to the reversible H-bond network between the amide groups. As a result, optimizing these intermolecular interactions is the key difficulty without compromising the other molecular characteristics of nylon polymer.

#### 3.1.4 UV–vis spectrum of nylons

**Fig. 6:**
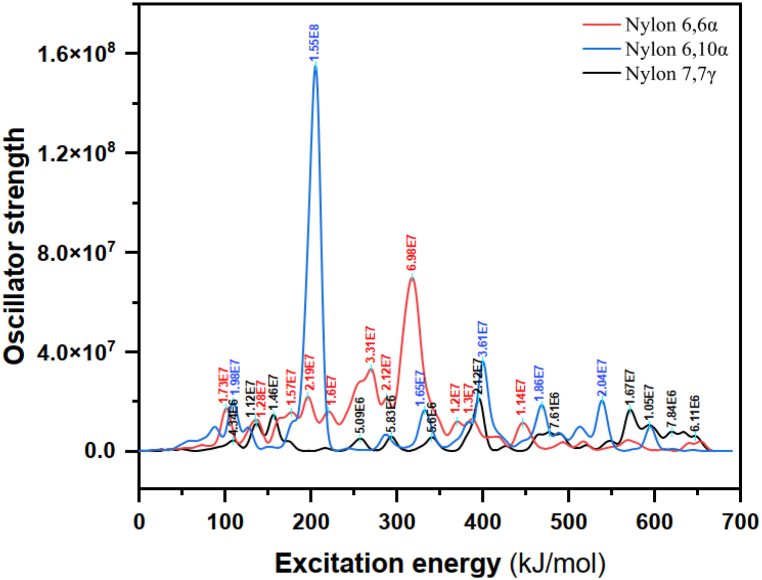
UV–Vis spectrum of the nylons: 66α, 610α, and 77γ.

UV light is used in polymerization processes to take advantage of the fact that molecules exposed to radiation produce reactive monomers in tiny axial deformation in their true form when subjected to temperature changes. The only function of UV radiation, according to conventional wisdom, is to initiating polymerization. Photo-excited structures, however, might take part in or perhaps be restricted to propagation. Fig. 6 depicts the UV–vis spectra of nylon polymers.

**Fig. 7:**
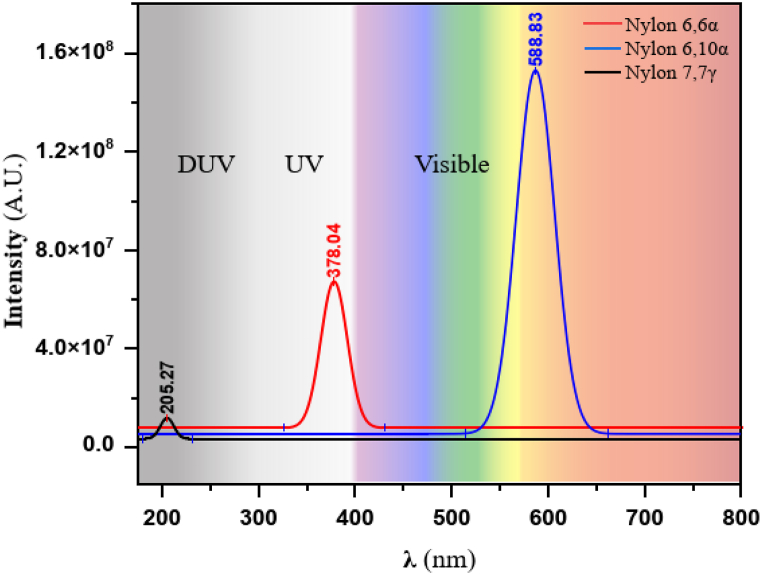
A spectral region for nylons: 66α, 610α, and 77γ.

The greatest peaks of excitation energy for nylons 66α,610α, and 77γ are 317.304, 205.279, and 395.382 kJmol^-1^, respectively, according to Fig. 6 and Table 5. Thus, according to the Planck equation [75], the simulated energies are inversely proportional to wavelengths. Fig. 7 displays, for three structures, the electroluminescence intensity at various wave lengths following Gaussian fitting. As can be seen, the peak around 589 nm is related to the orange-red visible light (Vis,400–800 nm) emitted from nylon 610α, the peak around 378 nm is related to the ultraviolet light (UV, 300–400 nm) and the peak around 205 nm is related to the ultraviolet light (DUV, 200–300 nm) emitted from nylon 66α, and nylon 77γ, respectively. As a result, it may be said that the entire spectral region contained gaps and edges.

**Table 5:** Excitation energy and Oscillator strength for polyamide nylons.

Nylon 66α		Nylon 610α		Nylon 77γ	
Excitation Energy *[kJmol*^*−*^*^1^]*	Oscillator strength	Excitation energy *[kJmol*^*−*^*^1^]*	Oscillator strength	Excitation energy *[kJmol*^*−*^*^1^]*	Oscillator strength
101.155	1.728x10^+7^	109.300	1.980 x10^+7^	109.441	4.341x10^+6^
220.364	1.602 x10^+7^	205.279	1.551x10^+8^	257.023	5.095 x10^+6^
317.304	6.979x10^+7^	332.368	1.652 x10^+7^	340.697	5.598 x10^+6^
387.388	1.296 x10^+7^	399.885	3.612 x10^+7^	395.382	2.117x10^+7^
446.338	1.142 x10^+7^	468.725	1.863 x10^+7^	465.878	6.824 x10^+6^
516.798	3.842 x10^+6^	538.889	2.042 x10^+7^	520.563	2.365 x10^+6^

An excited state is the outcome of an energetically preferred electron promotion from the highest occupied molecular orbital (HOMO) to the lowest unoccupied molecular orbital (LUMO) at energies in the 200–800 nm spectrum [76]. As a result, nylon 77 is capable n–σ* transition state; these transitions usually need less energy than σ–σ*. Therefore, nylon 66 transition falls in an UV region; this transition needs an unsaturated group in the molecule to provide π electrons, this effect also influences n–π* transitions but is overshadowed near the blue region, and for nylon 610 is capable π–π* transitions while the π–π* transitions are relatively localized to the amide group. The shift of the π–π* absorption peak to longer wavelengths may be caused by hydrogen bonding [77]. Therefore, using monomer and dimer models, this investigates the impact of hydrogen bonding. Fig. 7 illustrates the structures of nylon dimer models with the calculated wavenumbers, as well as electronic transitions involving σ electrons in various molecules and π–π* transitions in molecules such as amides and nylons**.**

**Fig. 8:**
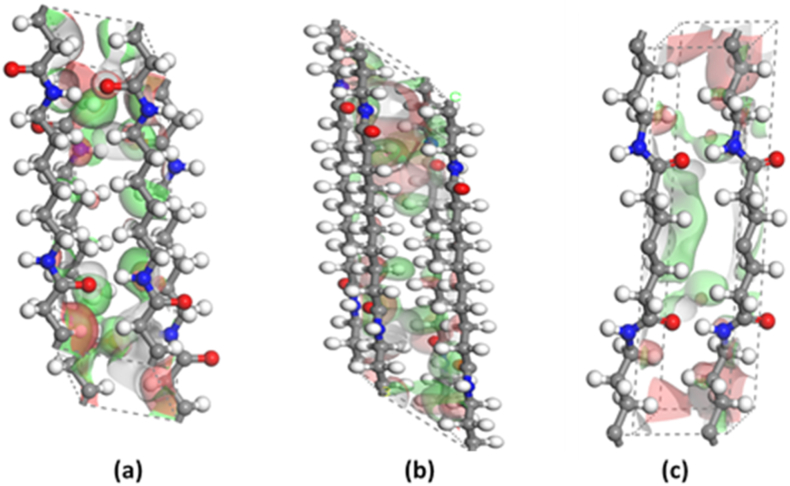
Highest and lowest molecular orbital electronic distribution for nylons: (a) 66α (b) 610α, and (c) 77γ.

As can be seen in Fig. 8(a–c), the hydrogen bonding that is attached to an atom with a high electronegativity has a low LUMO because there is a poor energy match between the two atoms, which results in a low splitting. The N, O lone pair has a high HOMO since they are not bonding; therefore, they can form a new bond. The hydrogen bonds were perfectly constituted by inverting alternate chains in the α-form of the nylon 66 model, while in the α-form of nylon 610 the progressive shear structure was adopted. These dimer models depicted the hydrogen-bonded structure of the π–π* transitions. The π–π* transitions were delocalized by being coupled with each other, and the oscillator strengths were accumulated in the highest state, while in nylon 66, the n–π* transitions were localized and the oscillator strengths (OS) were distributed to more than one state.

We found that core-to-bound and σ-to-continuum transitions must be stopped for any electrical structure-based method that attempts to concentrate on oscillator in the UV–vis region to be effective. To disrupt the molecular electronic structure, such techniques might employ customized electromagnetic fields [78], structural redesign of chromophores and chromophore assemblies, and [79,80] customized molecules-nanostructure assemblies.

Radiative recombination is poor in indirect bandgap materials, which also means that it will often account for a smaller proportion of total recombination than the non-radiative recombination that occurs at point defects or grain boundaries, which accounts for the majority of recombination. Radiative recombination is ultimately required for the excited electrons to return to the valence band if they are unable to reach these recombination spots [81]. The polymer can be made to create a dislocation loop to do this. The bandgap is said to be “direct” when electrons and holes share the same crystal momentum in the conduction and valence band, allowing an electron to produce a photon directly.

**Fig. 9:**
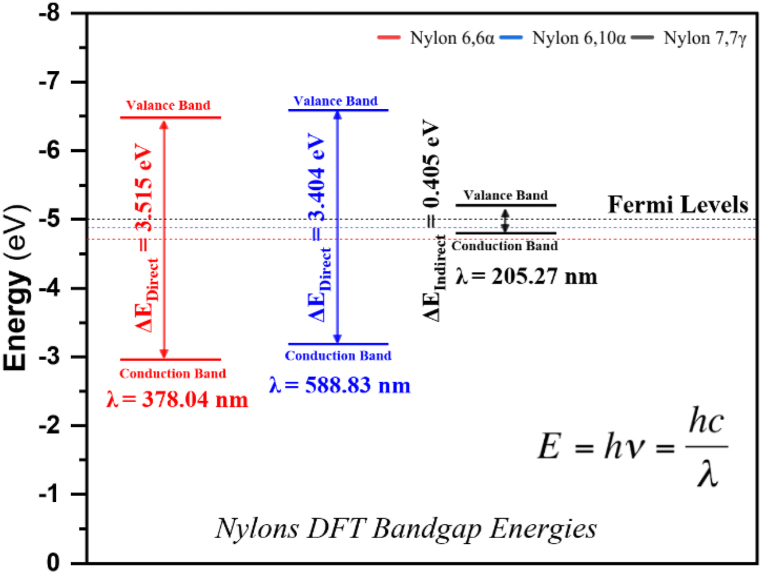
DFT Bandgap energies for nylons: 66α, 610α, and 77γ.

For nylon 66α and 610α, the direct Density Functional Theory (DFT) bandgap is inversely proportional to the wavelength, as seen in Fig. 9. Therefore, a photon cannot be emitted in an "indirect" gap for nylon 77γ as the electron must transit through an intermediary state and transfer momentum to the crystal lattice. The behavior of the electron transitions in polymer structures depends on the temperature dependence of the fermi level, which is located between the valence and conduction bands. As a result, electrical and thermal properties are determined using fermi energy. Table 6 shows the valence and conduction band energies for the three nylon 66α, 610α, and 77γ. Since the band energy gaps of nylon 66α and 610α are closer and higher than those of nylon 77γ, these two types of nylon are suspected to be more resistant to chemical alterations than nylon 77γ. Additionally, it demonstrates that these two nylons are more stable and possess superior insulating characteristics. Therefore, the nylon 66α and 610α polymers can be selected first, followed by the nylon 77γ, when the insulation performance of the enameled wire insulating is relatively high.

**Table 6:** DFT (E_*g*_) bandgap energies and Fermi Levels for polyamide nylons.

Polymer	Valance band edge [eV]	conduction band edge [eV]	DFT ΔE_*g*_ [eV]	Fermi Level [eV]
**Nylon 66α**	−6.478	−2.963	3.515 direct	−4.721
**Nylon 610α**	−6.591	−3.187	3.404 direct	−4.889
**Nylon 77γ**	−5.203	−4.798	0.405 indirect	−5.001

### 3.2 Thermodynamic characteristics

#### 3.2.1 Heat capacity

**Fig. 10:**
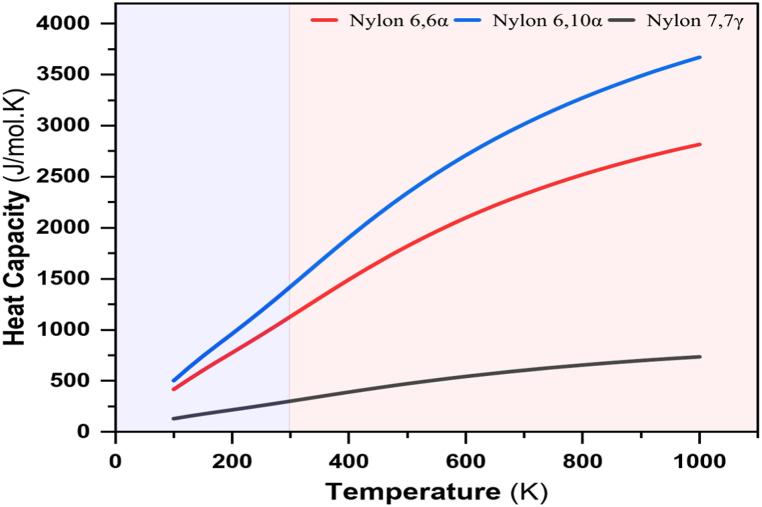
Heat capacity vs Temperature behavior of nylons: 66α, 610α, and 77γ.

A crucial thermal attribute is heat capacity. It provides concrete experimental evidence of the molecular and component mobility. However, measurements derived from it, such as enthalpy, entropy, and Gibb's free energy, can provide vital information regarding the state of the polymer. Quantitative C_p_ is crucial from a technological perspective as well. The above-mentioned values' temperature dependency is particularly important in semicrystalline polymer melting and crystallization processes since they frequently take place over a large temperature range. Investigating glass transition events that take place in more or less amorphous polymers takes this into account as well. C_p_ represents the heat capacity as a function of temperature whether it is amorphous or molten. The heat capacity of nylon polymers was evaluated between 100 and 1000 K. Particularly; a major source of heat capacity is the breakdown of hydrogen bonds when temperature rises. Therefore, a change in heat capacity during a process will be reflected in a change in the net extent of hydrogen bonding. The results in Fig. 10 show how the heat capacity fluctuates with temperature.

As shown in Fig. 10, the vibrational motion in the solid state [56] describes the heat capacity below the amorphous nylon 66's reported glass transition at 323K. The molar heat capacity of nylons was also roughly predicted by polynomial regression equations:

$$C_{p_{Nylon66\alpha }}\left(T\right)=-12.65009+4.01437T-4.56{*10}^{-4}.T^{2}-7.79*10^{-7}.T^{3}$$

$$C_{p_{Nylon610\alpha }}\left(T\right)=-33.32221+5.04277T-2.42{*10}^{-4}.T^{2}-1.21*10^{-6}.T^{3}$$

$$C_{p_{Nylon77\gamma }}\left(T\right)=30.24594+0.92084T+6.45{*10}^{-6}.T^{2}-2.36*10^{-7}.T^{3}$$

As seen in Fig. 10, the values of heat capacity at room temperature for nylons 66α, 610α, and 77γ are 1123.03, 1416.57, and 299.10 Jmol-1 K^−1^, respectively. Therefore, from the experimental literature data of 2022 [82], the average heat capacity values are 490.80, 615.00, and 145.73 Jmol-1K^−1^, respectively. The amounts of heat required with increasing the temperature of substituted nylons 66α and 610α are more significant than that for nylon 77γ. The polyamide becomes more mobile and changes structurally from rigid to flexible, which alters the polymers' ability to withstand heat. The literature has already reported on these phenomena [83,84]. Homologous series of polymers exhibit more pronounced increases in heat capacity with temperature in liquid chain segments having more H atoms due to the persistent excitation of the high-frequency C–H, N–H, and O–H stretching vibrations. Due to the fact that at higher temperatures, the regular modes of the heavy atoms' stretching vibrations and the light and heavy atoms' bending frequencies are usually already activated. In the absence of numerous H-atoms, a decreased C_p_ in the liquid state is feasible because the chain atoms' torsional vibrations transform into hindered rotors, which reduces their contribution to C_p_ [85]. However, the C_p_ of the crystalline nylon 66 when approaching the Brill transition temperature exceeds the C_p_ of the liquid, that is, before reaching the pseudo-hexagonal crystal structure, rising disorder happens without an abrupt phase transition, and therefore it must contain additional, reversible entropy contributions.

#### 3.2.2 Glass transition temperature

**Fig. 11:**
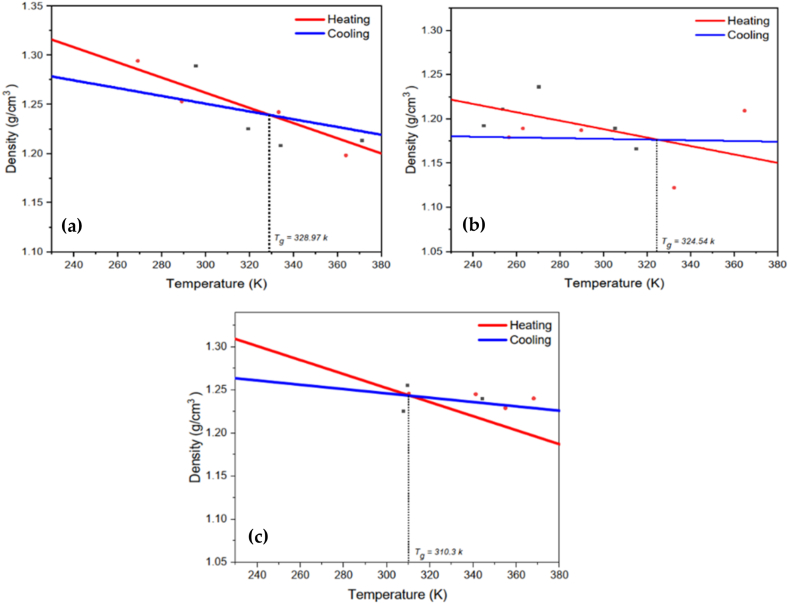
Glass transition temperature for Nylons: (a) 66α (b) 610α (c) 77γ.

Crystallites are made up of stacks of crystalline sheets in the triclinic -phase of the most stable PA66 crystalline structure. Each thermogram shows an exothermic peak that precedes the melting peak at 265 °C. This peak was attributed to a fresh sub-melting temperature transition, which Khanna had previously detected in PA 6, PA 66, and PA 12. In the literature, the development of this exotherm is attributed to rapid cooling of polymers [[86], [87], [88]]**.** The more stable α-phase is developed upon slow cooling, and the chains are organized in a triclinic unit cell. A pseudohexagonal γ-mesophase is formed when the melt and is only metastable at lower temperatures [89]. This γ-mesophase heats up and permanently changes into the α-phase [90]. Micro indentation hardness values can be used to distinguish changes in the crystalline lattice of a polymer [[91], [92]]. Up until the glass transition temperature, the first coefficient can be connected to the thermal expansion coefficient of polyamide structures. The second one, however, is related to the thermal expansion coefficient of the rubbery substance. The temperature dependence of the polymer's density is used to calculate the glass transition temperatures, Tg. Fig. 11(a) illustrates nylon 66α glass transition temperature Tg nylon 66α = 328.97 K. Therefore, Fig. 11(b and c) the nylon 610α and 77γ glass transition temperature Tg nylon 610α = 324.54 K and Tg nylon 77γ = 310.30 K, respectively.

It is essential to mention that each data set corresponds to the mean value of three polymers. It should be noted that these polymers' behaviors were comparable. Due to the transition in semicrystalline polymers broadening, the glass transition temperature, T_g_, shifts from 323 K for amorphous nylon 66–332.7 K when taken at the midpoint of the increase in C_p_ [85]. In addition, data on the regulation of nylon 66, the degree of crystallinity, and the glass transition temperature [93]. Polymer may undergo breakdown processes at higher temperatures after melting, and the glass transition temperature, T_g_ = 323 K, and the Brill transition temperature occur between 170 °C and 220 °C, well below the Tm, which is in the range of 250–272 °C. Fig. 11(a) implies nucleation primarily occurs at different temperatures <330 K before crystal growth dominates at higher temperatures. In consideration of the cooling rates used in this research, the T_g_ of PA66α is about 329 K; as a result, the aforementioned data is consistent with the conclusion that homogeneous nucleation is more effective at temperatures just below T_g_ [94]. Later, taking into account how the corresponding enthalpies alter with temperature, further specifics concerning these processes will be discussed.

**Fig. 12:**
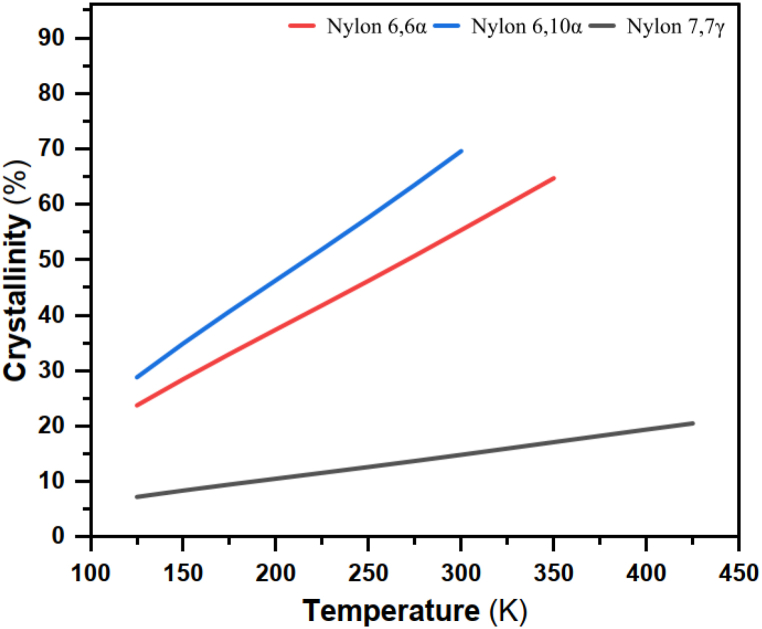
Crystallinity and temperature for the nylon polymers.

According to ASTM E1269, nylon 77 has a substantially lower glass transition temperature than nylon 66 α and nylon 610 α, which is understandable given that it has a lower glass transition temperature (Tg = 310.3 K). It could be correlated to the higher crystallinity in nylon 610α due to the nucleation effect and the lower crystallinity in nylon 77γ, probably due to the interruption of the H-bonding structure, as seen in Fig. 12. In order to decrease the damage caused by loading, we anticipate that the energy from the H-bond interactions will increase. Since nylon 66α is a semicrystalline polymer composed of a rigid crystalline phase and an amorphous phase in which void nucleation exclusively occurs, it makes sense to assume that altering the inter-chain interactions in the amorphous phase would have a large impact on nylon 66 α 's durability.

**Fig. 13:**
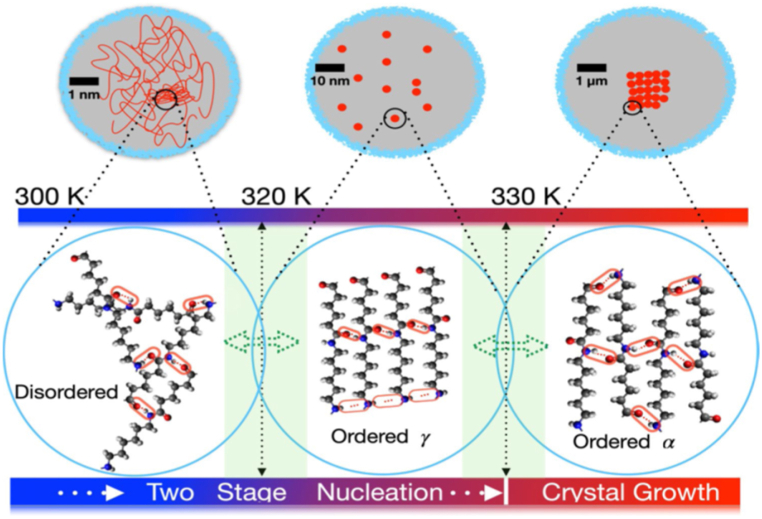
Schematic representation of the evolution of the PA66 from the quenched state through nucleation to crystalline state.

Any γ -phase that is trapped during sample cooling is likely to have a more refined structure and a stronger hydrogen bond than if it had formed during sample heating. In contrast, the variations in the melting endotherms of the γ-phase are typically negligible. They do not appear to indicate that the nylon 77γ structure contains any significant γ-phase fraction prior to heating. Fig. 13 shows the triclinic α polymorph of PA66 has an anti-parallel chain structure [95].

Following are some observations: (a) A broadened transition of the semicrystalline sample is observed, beginning at the glass transition temperature of the mobile-amorphous phase (T_g_ = 323 K) and reaching 342 K (T_g_ = 332.7 K), an extra rigid amorphous phase (RAF) undergoes a separate, broad glass transition (340–400 K, T_g_ = 370 K). (b) The RAF transition, in turn, overlaps with the increasing large-amplitude motion of the CH_2_ groups within the crystals and latent heat effects from melting, recrystallization, and crystal annealing. (c) Due to additional entropy (disordering) contributions, the heat capacity of the crystals gradually exceeds that of the melt from 390 to 480 K. The heat capacity drops to the melt level above 440 K, close to the Brill temperature. (d) If an observation (c) is confirmed, there is some locally reversible melting on the crystal surfaces. (e) The increasing large-amplitude motion is described as a crystal glass transition.

The entire view of the changes that take place when semicrystalline nylon is heated from the solid to the liquid state has been revealed by this research. Skeletal and group vibrations are excellent representations of the molecular motion below the glass transition of the mobile-amorphous fraction (T_g_ = 323 K) [96]. As the low-temperature T_g_ is approached, torsional oscillations progressively change into internal rotations, causing large-amplitude motion to get excited. T_g_ shifts to 332 K when crystals are present, and its magnitude is diminished when a rigid-amorphous percentage of the semicrystalline polymer is present.

#### 3.2.3 Enthalpy

The VAMP model was used to calculate the enthalpy values using single-point energy calculations. In the single-point energy calculation, the wave function, charge density, and subsequently the energy for a specific molecule with a predetermined geometric configuration are all calculated. Based on this single-point energy estimate, the VAMP model employs semi-empirical computations [97]. As a result, using equation (2), the enthalpy values were used to calculate the crystallinity rate, as shown in Fig. 12:

(2) $$\%Xc=\frac{\Delta H}{\Delta H_{o}}$$

Where: %Xc: crystallinity rate, ΔH represents experimental enthalpy (kJmol^−1^), and ΔH° is the theoretical melting enthalpy (kJmol^−1^); ΔH° for 66α, 610α, and 77γ are 188.4, 197.0, and 192.7 Jg^-1^, respectively [98,99].

**Fig. 14:**
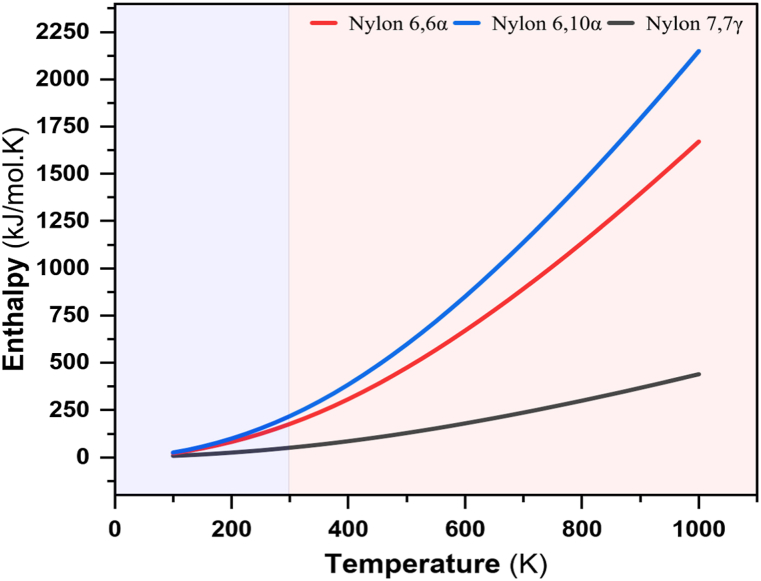
Experimental enthalpies vs temperatures for polyamide nylons.

The experimental value of ΔH, as seen in Fig. 14 and Table 7 for nylon 66α between simulated glass transition temperature and room temperature is 35.077 kJmol^-1^; this demonstrates that excellent agreement was achieved by Domalski et al. (1986) is 165.3 Jg^-1^ (37.41 kJmol^-1^), and for annealed nylon 66α is 139.3 Jg^-1^ (31.53 kJmol^-1^) [100]. Therefore, Matsumura et al. (1999) from experimental data of PVT measurements is 28.1 Calg^−1^ (26.63 kJmol^-1^), and from the literature, data is 45 Calg^−1^ (42.64 kJmol^-1^) [101]. The values of X_csimulated_ from Fig. 12 are depicted at 300 K are, for nylon 66α at is 55.34 %. Furthermore, the experimental value of ΔH for nylon 610α is 40.252 kJmol^- 1^. The crystallinity rate X_csimulated_ value for nylon 610α is 69.59 %. Therefore, the experimental value of ΔH for nylon 77γ is 1.438 kJmol^-1^, and the value of X_csimulated_ is 14.81 %.

**Table 7:** Experimental enthalpies for polyamide nylons.

Polyamide	H_Exp.@ 298.15 K_	H_Exp.@ TgSim._	ΔH_Exp._
	[kJmol^−1^]	[kJmol^−1^]	[kJmol^−1^]
**Nylon 66*α***	175.495	210.572	35.077
**Nylon 610*α***	216.897	257.149	40.252
**Nylon 77*γ***	51.288	52.726	1.438

This indicates the high agreement Marset et al. (2020) were able to obtain in relation to the melting process for nylon 610 α. The melting enthalpy, 62.9 Jg-1 (17.76 kJmol- 1), allows the calculation of the degree of crystallinity (Xc) of PA610, with a value of 32 % [102]. Wu et al. (2018) reported similar results, who showed an Xc of 34 % [103].

The higher enthalpy in nylon 610α indicates the presence of hydrogen bond-maintained clusters, which have higher dissociation energies. At the glass transition temperature, or 257.15 kJmol^-1^, the hydrogen bond strength is still extreme, but this has no impact on the polymer's subsequent recrystallization. Although the hydrogen bonds are still very strong, the unlimited content of the ordered chain aggregates is probably too low. The shortest H-bonding is 2.102 Å, and the average H-bonding distance for nylon 77γ is 2.153 Å, so a meaningful self-nucleation effect is not noticed. The thermodynamic constants ΔH derived from models of nylon structures are linearly proportional to the temperature rate. The enthalpy significantly depends on the structure of the dimeric adduct as well. If a structure is obtained, as proposed for nylon 66α, the magnitude of the thermodynamic parameters per hydrogen bond is higher than for a linear dimer such as nylon 610α and 77γ. Vasanthan, N. (2009) showed that variations in crystal form from γ to α depending on thermal and mechanical treatment were responsible for increases in polyamide crystallinity [104]. The melting of microcrystals formed in the amorphous region has been attributed to some authors [[105], [106]]. Since the samples are aged at a temperature above T_g_, the formation of these micro-crystallites can be attributed to a post-crystallization effect. Structure modifications during post-crystallization can take the form of chain rearrangements brought on by hydrogen bond breaking, tie-molecule relaxation, and accelerated chain motion [66]. According to Shu et al., as the chain motion increases, mobile chains in the amorphous region can combine with the crystallites, increasing the crystallinity [107]. In order to more fully comprehend the impact of chain length and assess how this affects the potential for enantioselective crystallization, Davey et al. [108] did calculations using molecular modeling. Therefore, the enthalpies of dimer production have been taken into account, and calculations are being done on them. The ΔH stabilization is derived by this means of subtracting the summation of the single molecules from that of the ΔH dimer molecules**.**

#### 3.2.4 Entropy

The increase in entropy must overcome the handicap of an endothermic process so that*:*

(3) TΔS>ΔH

Since the effect of the temperature is to "magnify" the influence of a positive ΔS, the process will be spontaneous at temperatures above ΔH > 0 and ΔS > 0. This can be described through Equation (4) as the following:

(4) $$T=\frac{\Delta H}{\Delta S}$$

**Fig. 15:**
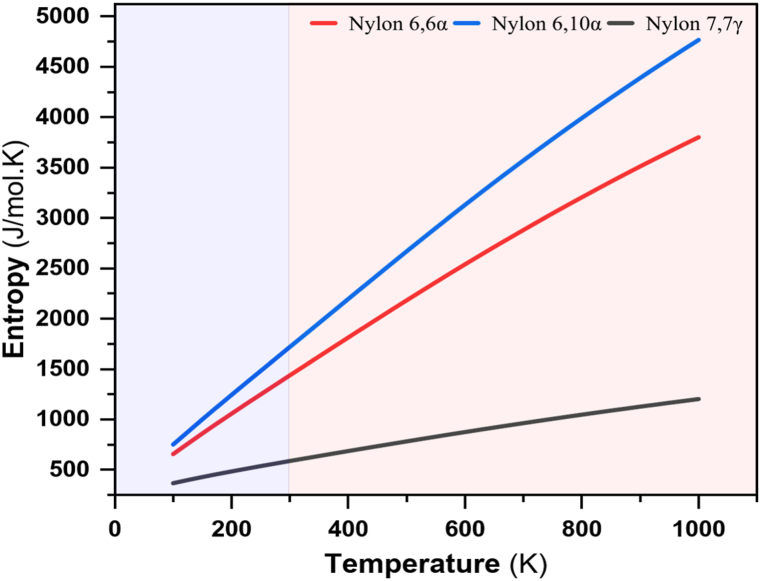
Experimental entropies vs temperatures for polyamide nylons.

The experimental values of ΔS for nylon 66α, 610α, and 77γ between simulated glass transition temperature and room temperature are 113.749 Jmol^-1^, 128.843 Jmol^-1^, and 15.095 Jmol^-1^, respectively, as seen in Fig. 15 and Table 8. The values of these polymers indicated endothermic and spontaneous process. These values are in good agreement with the typical values reported in the literature [109]. It is probable, however, that the values for ΔH and ΔS for other types of polyamides will not be greatly different from those for caprolactam polymers, provided the carboxyl and amino groups are attached to a straight chain of several CH_2_ groups. This is so for the important nylon 66, from adipicacid and hexamethylenediamine.

**Table 8:** Experimental entropies for polyamide nylons.

Polyamide	S_Exp.@ 298.15 K_	S_Exp.@ TgSim._	ΔS_Exp._
	[Jmol^−1^]	[Jmol^−1^]	[Jmol^−1^]
**Nylon 66*α***	1431.687	1545.436	113.749
**Nylon 610*α***	1708.241	1837.084	128.843
**Nylon *77γ***	583.158	598.253	15.095

A comparison is made between nylon 66α, 610α, and 77γ; Entropy significantly rises along with the formation of the amide group, which is most likely caused by the release of orientated water molecules bound by the charged carboxyl and amino groups. The formation of the amide group appears to involve an unusually large increase in disorder 113.749, 128.843 and 15.095 Jmol^-1^ for nylon 66α, 610α, and 77γ, respectively. This may be interpreted in the following way. The charged carboxyl and ammonium groups hold around themselves several oriented water molecules. The order is lost when the charged centers vanish, and at high temperatures, this causes an increase in entropy. The zones of influence of the charged groups in the a-amino-acids overlap, preventing the full manifestation of this action. The charged groups are sufficiently separated in the higher peptides and higher homologues of amino acids to operate independently.

#### 3.2.5 Free energy for polyamide nylons

The thermodynamic parameters ΔH, ΔS and ΔG for the hydrogen bond breaking process in nylons are substantially higher than was expected for hydrogen bonds in model amides. We have examined the model compounds diamide and diacid to see if our results were particular to the polymer. Each time, the hydrogen bond breaking process was observed above the melting point and a totally hydrogen bound spectrum was obtained at low temperatures. The model structure previously described was used in the data analysis, which was solely based on the NH stretching band. Entropy and enthalpy are two of the key components of free Gibb's energy, and as such, they have a significant influence on the outcome when interpreting the data from the aforementioned figures. While the partition function is used to determine the entropy and enthalpy terms, Gibb's free energy is instead calculated as shown in Equation (5) [110]:

(5) ΔG=ΔH−TΔS

The decrease in free energy provides the driving force for crystallization. At the equilibrium melting temperature T°_m_, the free energy of the melt and the crystal is equal (ΔG_*f*_ = 0). Therefore, the entropy of fusion at T°_m_ can be expressed as in Equation (6):

(6) $$\Delta Sf=\frac{\Delta Hf}{T{}^{\circ}m}$$

**Fig. 16:**
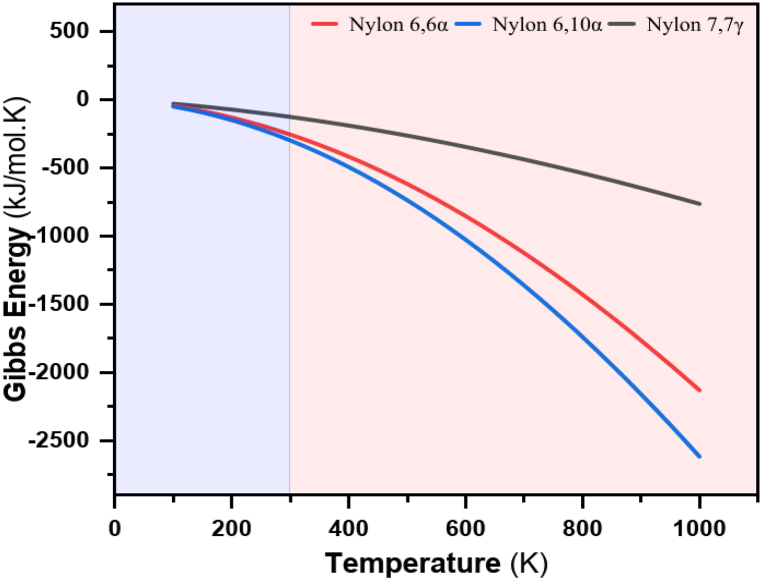
Gibb's Free Energy for polyamide nylons.

Even though the driving force for crystallization is the free energy difference, it does not always occur immediately, especially in the early stages of crystallization (nucleation). This is due to the potential for the surface tension of the newly formed crystal (nucleus) surface tension to increase free energy. Fig. 16 displays the relative experimental and simulated Gibb's energies taken from the polymer curve. Li et al. (2020) [111] discovered that a high H-bond density favors fast crystallization at higher temperatures due to thermodynamic driving forces.

Table 9 displays the outcomes of such calculations for all thermodynamic parameters for which the required data are available.

**Table 9:** Experimental Gibb's energies from the curve for polyamide nylons.

Polyamide	ΔH_Exp._	ΔS_Exp._	G_Exp.@ 298.15 K_	G_Exp.@ TgSim._	ΔG_Exp._
	[kJmol^−1^]	[Jmol^−1^]	[kJmol^−1^]	[kJmol^−1^]	[kJmol^−1^]
**Nylon 66*α***	35.077	113.749	−254.617	−299.182	−44.565
**Nylon 610*α***	40.252	128.843	−295.698	−342.320	−46.622
**Nylon *77γ***	1.438	15.095	−125.207	−132.063	−6.856

Since both ΔH and ΔS are functions of temperature, if phase transitions do not occur, the enthalpy and entropy are typically not extremely temperature dependent. As a result, it is typically accurate enough to function with values provided at a specific temperature, such as 25 °C. Since both ΔH and ΔS changes with temperature, the positive value of ΔS suggests an increase in disorder and for nylon 66α and nylon 610α their ratio changes only slightly. The thermodynamic parameters obtained between the room and glass transition temperatures are shown in Table 9, the results of enthalpies and entropies are positive values where the values of Gibb's energies for nylon 66α, 610α, and 77γ are calculated as −44.565, −46.622, and −6.856 kJmol^-1^, respectively. Due to the distinct atomic/molecular structure, continuous growth process is noticed for rough contact; lateral growth process is typically observed in flat interface (Porter & Easterling 2001) [112]. This indicates that the process is spontaneous and exergonic at high temperatures. The differing magnitudes of the free energy reduction during the phase transition may have led to this rough/flat boundary. Another important observation is the temperature dependence of enthalpy and entropy fluctuations and their general impact on Gibb's free energy: increased relative enthalpy and entropy absolute values are a result of the temperature rise. The relative Gibb's free energies of intermediate structures for the polymers increase as the temperature rises; however, the increase is greater for nylon 610α. Importantly, the increase in relative Gibb's free energy follows the increase in temperature. Structures' relative Gibb's free energy hardly alter when the temperature rises. As anticipated, the process occurs spontaneous as a result of the temperature increase alone.

#### 3.2.6 Thermal expansion coefficient (CTE)

Some significant thermal characteristics, like the glass transient temperature and the coefficient of thermal expansion, are necessary in order to study the thermal-mechanical behavior of nylon polymers. The dimensional stability of polymers is shown by their coefficient of thermal expansion, which is a crucial feature. This characteristic is typically influenced by temperature, polymer composition, manufacturing method, and form [113]. The coefficient of thermal expansion for polyamides is only known for a select few temperature ranges [114,115]. Additionally, the polymers' thermal expansion and contraction can be influenced by the geometry, crystalline structure, and thermal history of the materials [116].

**Fig. 17:**
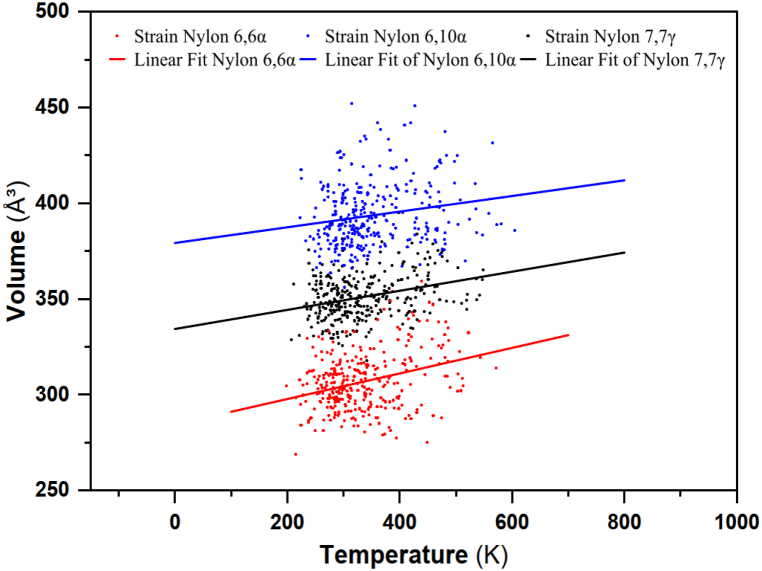
Thermal expansion coefficient for nylon polymers.

Thermal expansion behaves differently when the temperature is constant. For instance, the thermal deformation of a polyamide polymer at a fixed temperature is a function of time. These numbers were taken directly from Fig. 17 while only taking into account the range of isothermal circumstances. This Figure illustrates how the thermal expansion tends to cap its value. The results of thermal deformation obtained with both methods can be used to estimate the thermal expansion coefficient and the transition temperature of the polyamide. From the volume of the first data point (Vo) and the slope of the linear fit curve, the volumetric thermal expansion coefficient (αV) as expressed in equation 7:

$$y_{Nylon66\alpha }=284.37593+0.06674\;x$$

$$y_{Nylon610\alpha }=379.20593+0.04094\;x$$

$$y_{Nylon77\gamma }=334.29333+0.04985\;x$$

(7) $$\alpha V=\frac{1}{V_{0}}\;\frac{\partial V}{\partial \text{Tp}}$$

We can then use equation (8) to determine that the coefficient of linear thermal expansion CTE (α_l_) is approximately:

(8) $$\alpha l\approx \frac{\alpha V}{3}$$

The simulated expansion coefficients of nylon 66α, nylon 610α, and nylon 77γ are 7.33 × 10^−5^ K^-1^, 3.38 × 10^−5^ K^-1^, and 4.36 × 10^−5^ K^-1^, respectively. When the data for nylon 66 is compared to that for nylon 610 and nylon 77, it is clear that nylon 66 has a significantly higher thermal expansion coefficient. Kim et al. (2019) [117] developed a method to evaluate thin films' thermal expansion freely on water. However, when both ends of a polyamide fiber sample are free, incorrect strain values can be produced due to bending effects. Samples that are completely constrained are not even allowed to be used. It is necessary to create alternative methods with high accuracy and precision in order to characterize the nonlinear thermos-mechanical behavior of polyamides in their actual structural form, despite the existence of both standard and novel approaches. Moran et al. (2016) [118] reported similar degradation parameters for PA610, where the degradation temperature was above 440 °C. These results are very interesting since they indicate the PA degradation occurs at high temperatures compared to commodities and some engineering plastics.

## 4 Conclusion

The results of this study are mainly attributable to the influence of thermal expansion coefficient and thermodynamics on the structure of nylon 66α, 610α, and 77γ were examined in terms of their structural characteristics of structures tested at various temperatures. The study has produced various findings.

• The results indicated that the modeled nylons crystal structures have excellent thermodynamic characteristics, and the kinetic energy sinusoidal wave means values for nylon 66α, nylon 610α, and nylon 77γ were 154.92, 214.93, and 163.11 kJmol^-1^, respectively. From these results, it is clear that the highest kinetic energy for nylon 610α with a value of 214.93 kJmol^-1^; therefore, the simulated expansion coefficients of nylon 66α, nylon 610α, and nylon 77γ are 7.334 × 10^−5^ K^-1^, 3.38 × 10^−5^ K^-1^, and 4.36 × 10^−5^ K^-1^, respectively. When the data for nylon 66α is compared to that of nylon 610α and nylon 77γ, it is clear that nylon 66α is a promising material for usage as fibers and engineering thermoplastics due to its much greater thermal expansion coefficient.
• The enthalpy ΔH of nylon 66α and nylon 610α demonstrated higher values, 35.08 and 40.25 kJmol^-1^, compared to nylon 77γ 1.44 kJmol^-1^, and these positive values of ΔH reflect the endothermic processes. The change of entropy values ΔS, for nylon 66α and nylon 610α, are near the same magnitude and lie within the range +113.75 to +128.84 Jmol^-1^, all with positive signs. Therefore, a more random molecular configuration confirmed that the structures proceeded in relatively similar mechanisms, comparing by nylon 77γ the change of entropy values ΔS is 15.10 Jmol^-1^. Tracking total energy changes as a system response to the temperature is possible thanks to the impact of the rising temperature on the thermodynamic profile of the structures.
• Covering the crystallinity and glass transition temperatures in the examined temperature range is crucial for polymers. Fig. 16 and Table 9 show how temperature affects the thermodynamic characteristics of nylon polymers and changes in relative Gibb's free energies. The values of Gibb's energies for nylon 66α, 610α, and 77γ are calculated as −44.57, −46.62, and −6.86 kJmol^-1^, respectively. That means the process is spontaneous and exergonic at high temperatures.
• It is necessary to establish alternative methods with high accuracy and precision in order to define the nonlinear thermodynamic behavior of polyamide nylons in their real form, despite the existence of both standard and novel approaches. The results of the molecular dynamics modeling and the experiments on the glass transition temperature, thermal expansion coefficient, and thermodynamic properties of nylon had good agreement. This led to the conclusion that aliphatic polyamide polymers characteristics might be adequately studied using molecular simulation.

## Funding

The author states that no funding was involved.

## Data availability statement

The data presented in this study are available from the corresponding author upon request.

## CRediT authorship contribution statement

**Ali F. Al-Shawabkeh:** Conceptualization, Data curation, Formal analysis, Investigation, Methodology, Software, Validation, Visualization, Writing – original draft, Writing – review & editing.

## Declaration of competing interest

The author declare that they have no known competing financial interests or personal relationships that could have appeared to influence the work reported in this paper.

## Nomenclature

ΔH_m_: Enthalpy of melting
ΔS: Entropy change
CG: Coarse-grained
DFT: Density Functional Theory
DIC: Digital Image Correlation
DSC: Differential scanning calorimetry
EM: Energy minimization
G: Gibb's energy
HOMO: Highest occupied molecular orbital
LUMO: Lowest unoccupied molecular orbital
M_n_: Molecular weight
MPD: *m*-phenylenediamine
MS: Molecular structures
MXD: 1,3-phenylenedimethanamine
PA: polyamide
PPDA: *p*-phenylenediamine
PVT: pressure-volume-temperature
RAF: Rigid-amorphous fraction
ReaxFF: Reactive Force-Field
SEM: Scanning electron microscopy
T_g_: Glass transition temperature
TGA: Thermogravimetric analysis
T_m_: Melting temperature
TS: Transition state
Χ*c*: Degree of crystallinity
